# Polysomnographic and nocturnal heart rate correlates of cognitive impairment in Parkinson’s disease: a cross-sectional study

**DOI:** 10.3389/fneur.2026.1913495

**Published:** 2026-10-09

**Authors:** Zhang Linwei, Lv Pu, Wang Lu, Zhang Xiangfei, Wang Kang, Jin Miao

**Affiliations:** Department of Neurology, China-Japan Friendship Hospital, Beijing, China

**Keywords:** cognitive impairment, nocturnal heart rate, Parkinson’s disease, polysomnography, REM sleep latency

## Abstract

**Objective:**

To investigate the correlations between cognitive impairment in Parkinson’s disease (PD) and nocturnal video-polysomnography (vPSG) and electrocardiogram (ECG) parameters, evaluating their independent clinical value.

**Methods:**

We analyzed 84 PD patients divided into normal cognition (PD-NC, *n* = 27), mild cognitive impairment (PD-MCI, *n* = 27), and dementia (PDD, *n* = 30) groups. Clinical scales, overnight vPSG, and electrocardiogram parameters were recorded. Multivariable linear regression was used to evaluate independent factors associated with the Montreal Cognitive Assessment (MoCA) scores.

**Results:**

As cognitive impairment progressed, REM sleep latency significantly shortened (128.82 ± 117.32 min in PDD vs. 200.54 ± 90.26 min in PD-NC, *p* = 0.006). Concurrently, mean nocturnal heart rate (65.80 ± 10.80 bpm) and minimum heart rate (50.77 ± 12.51 bpm) were significantly higher in the PDD group than in the other two groups (*p* < 0.01). Multivariable linear regression revealed that, after adjusting for age, education, and UPDRS III score, mean nocturnal heart rate remained independently and negatively associated with MoCA scores (*B* = −0.158, *β* = −0.264, *t* = −3.018, *p* = 0.003), while REM sleep latency was independently and positively associated with MoCA scores (*B* = 0.010, *β* = 0.192, *t* = 2.224, *p* = 0.029). Collinearity diagnostics showed no significant multicollinearity (*VIF*: 1.029–1.261).

**Conclusion:**

Higher mean nocturnal heart rate and shorter REM sleep latency were independently associated with lower global cognitive performance in PD patients. Both measures can be obtained from routine overnight PSG, although their physiological significance remains uncertain. The cross-sectional design and the absence of direct autonomic or cholinergic measurements preclude causal or mechanistic conclusions.

## 1 Introduction

Parkinson’s disease (PD) is a rapidly growing neurodegenerative disorder worldwide. Its associated disability stems not only from classical motor signs but also from non-motor manifestations, most notably cognitive impairment and sleep disturbances (1, 2). Previous studies indicate that up to 30–80% of PD patients will develop Parkinson’s disease dementia (PDD) (1). This progression impairs daily functioning and increases the caregiving burden imposed on families and healthcare systems (1, 2). Identifying objective and sensitive biomarkers that reflect the decline of central cognitive networks is essential for early clinical intervention.

Overnight vPSG is the standard method for quantifying macro- and micro-sleep structures alongside associated neurophysiological events (3–5). Previous investigations have predominantly focused on macroscopic parameters such as reduced overall sleep efficiency, prolonged wakefulness after sleep onset, or diminished slow-wave sleep (6, 7). Comorbid obstructive sleep apnea has been associated with worse cognitive and motor outcomes, representing an important confounding factor in sleep-related biomarker research (8, 9). REM sleep microstructural alterations and their relationship to central cognitive decline are drawing increasing research interest (10). These central changes parallel α-synuclein–related degeneration of the peripheral autonomic nervous system in early PD (11). Nocturnal sleep, unperturbed by daytime environmental confounders, offers a robust physiological baseline for evaluating cardiac autonomic control in patients (12, 13).

Although sleep disturbances have been linked to cognitive impairment in PD, the independent associations of specific vPSG parameters with cognition remain unclear after adjustment for relevant clinical confounders. We therefore investigated the relationships between REM latency, whole-night summary heart-rate measures, and cognitive performance in 84 patients with PD using adjusted multivariable linear regression. Because beat-to-beat HRV and sleep-stage-specific ECG measures were not analyzed, the cardiovascular variables were considered nocturnal physiological correlates rather than direct measures of autonomic balance.

## 2 Materials and methods

### 2.1 Participants

A total of 84 patients with idiopathic PD who were admitted to or evaluated at the Department of Neurology at China-Japan Friendship Hospital between January and December 2024 were consecutively recruited. The clinical diagnosis of PD was established according to the Movement Disorder Society Clinical Diagnostic Criteria (14). This study received formal approval from the Institutional Review Board and Clinical Research Ethics Committee of the China-Japan Friendship Hospital (Approval No. 2023-KY-299), and informed consent was obtained from all patients or their legal proxies prior to study entry.

Inclusion Criteria: (1) Met the clinical diagnostic criteria for PD; (2) Completed a comprehensive battery of neuropsychological assessments; (3) Successfully completed standardized overnight vPSG with an effective total sleep time exceeding 4 h. Exclusion Criteria: (1) Concomitant neurodegenerative or structural central nervous system disorders; (2) Severe uncontrolled cardiac arrhythmias, severe respiratory failure, or severe obstructive sleep apnea (AHI > 30); (3) Severe visual, auditory, or psychotic deficits precluding cooperative cognitive testing; (4) Patients who had used benzodiazepines, non-benzodiazepine hypnotics, melatonin receptor agonists, sedating antidepressants prescribed primarily for insomnia, or sedating antipsychotics within 2 weeks before vPSG were excluded, antiparkinsonian medications and cardiovascular or autonomically active medications were not exclusion criteria.

### 2.2 Clinical and neuropsychological assessment

All clinical and behavioral evaluations were performed during the standardized “On-state” by two certified neurologists blinded to the patients’ vPSG profiles. Age, sex, disease duration, education years, height, weight, and BMI were systematically captured. Motor symptom severity was quantified using the UPDRS III, while disease staging was mapped via the H-Y scale.

Global cognition was examined via the MMSE and the MoCA. Neuropsychiatric and mood disturbances were tracked using Hamilton Anxiety/Depression Scales (HAMA/HAMD), while functional impairment in daily operations was indexed by activities of daily living (ADL). Adhering to the MDS task force guidelines (15–17) and Chinese consensus standards (18, 19), participants were classified into three operational cohorts: PD with normal cognition (PD-NC, *n* = 27), PD with mild cognitive impairment (PD-MCI, *n* = 27), and PD with dementia (PDD, *n* = 30). Specifically, PD-MCI was defined according to the MDS Task Force Level I criteria, which required a subjective or clinically observed cognitive decline, objective cognitive impairment on formal testing, and preserved functional independence. PDD was diagnosed according to the MDS clinical diagnostic criteria for dementia associated with Parkinson’s disease, characterized by significant cognitive impairment affecting multiple domains that directly interfered with daily functional activities.

### 2.3 Overnight video polysomnography and nocturnal heart rate assessment

Overnight vPSG was conducted using a 32-channel digital system (Nihon Kohden 9,200 K). Electroencephalogram signals were recorded from 16 channels referenced to linked mastoids (A1, A2) according to the International 10–20 system. Horizontal and vertical electrooculograms, electromyograms of the submental and bilateral anterior tibialis muscles, and a continuous ECG were simultaneously acquired. Airflow was monitored using nasal-oral thermistors and pressure transducers, chest and abdominal respiratory movements via pulse oximetry, and arterial oxygen saturation via pulse oximetry. Electrode impedance was maintained below 5 kΩ. Recordings commenced at the patients’ habitual bedtimes and concluded upon natural waking.

Sleep architecture was scored manually in 30-s epochs by two experienced sleep technologists blinded to clinical diagnoses, according to the American Academy of Sleep Medicine (AASM) scoring manual. Macro-sleep parameters and REM-specific variables were documented for analysis. Wakefulness was identified primarily by dominant posterior alpha activity, eye movements, and relatively high chin electromyographic tone. N1 sleep was characterized by attenuation of alpha activity with low-amplitude mixed-frequency EEG activity and slow eye movements. N2 sleep was identified by the presence of sleep spindles and/or K-complexes, whereas N3 sleep was defined according to the AASM slow-wave activity criteria. REM sleep was identified by low-amplitude mixed-frequency EEG activity accompanied by rapid eye movements and reduced chin electromyographic tone. REM sleep latency was defined as the interval from sleep onset to the first epoch scored as REM sleep. Disagreements between the two scorers were resolved by consensus.

Continuous single-lead ECG was recorded as part of the routine overnight vPSG protocol. The Polysmith system generated whole-night summary measures, including mean, maximum, and minimum heart rates. However, beat-to-beat RR interval series with standardized R-peak validation, artifact correction, and ectopic-beat removal were not generated as part of the routine PSG analysis. Consequently, time-domain, frequency-domain, nonlinear, and sleep-stage-specific HRV indices were not available for the present retrospective study.

### 2.4 Statistical analysis

Data analysis was performed using SPSS 30.0 and Python (statsmodels 0.14). The normality of continuous variables was assessed using the Shapiro–Wilk test. Continuous variables meeting the prespecified distributional assumptions were summarized as mean ± standard deviation. Differences among the three cohorts were assessed using one-way ANOVA followed by post-hoc LSD tests. Categorical variables were compared using the *χ*^2^ test. Because the clinical cognitive classification was ordinal, Spearman rank correlation analysis was used to screen associations between clinical, macro-sleep, nocturnal heart-rate parameters and cognitive impairment severity category.

Subsequently, multivariable analysis was executed using the continuous MoCA raw score as the dependent variable. Covariates known to dominate cognitive clinical presentations (age, education years, UPDRS III) along with the primary significant electrophysiological variables (REM latency, mean sleep heart rate) were entered into the model. Multi-collinearity was monitored via the Variance Inflation Factor (VIF), setting a strict threshold of VIF < 10. All statistical tests were two-tailed, with significance defined as *p* < 0.05.

## 3 Results

### 3.1 Demographic and clinical status across groups

A total of 84 consecutively recruited PD patients were included and stratified into three cognitive subgroups: PD-NC ( n=27 ), PD-MCI (n=27 ), PDD ( n=30 ). The demographic and baseline clinical characteristics are summarized in Table 1. No statistically significant differences were observed among the three groups regarding sex distribution (42 males / 42 females, $\chi^{2}=0.297$ , p=0.862 ), disease duration, BMI, or baseline anxiety and depression scores (all p>0.05 ). However, univariate analysis revealed that patients in the PDD group were significantly older and had fewer years of education compared to the PD-NC group ( p=0.028 ). Regarding clinical assessments, significant differences were identified across the groups in motor severity and cognitive function. Patients with PDD exhibited significantly higher UPDRS-III motor scores, higher H-Y stages, and elevated ADL scores compared to both the PD-NC and PD-MCI groups (all p<0.05 ). Furthermore, cognitive assessments demonstrated significant group-level differences in MoCA and MMSE scores ( p<0.001 ). *Post-hoc* pairwise comparisons confirmed that the PDD group had markedly lower MoCA and MMSE scores relative to both the PD-MCI and PD-NC groups (all adjusted p<0.05 ).

**Table 1:** Demographic and clinical characteristics according to cognitive impairment severity category.

Variable	PD-NC (*n* = 27)	PD-MCI (*n* = 27)	PDD (*n* = 30)	*χ*^2^/*F*	*p*
Age, years	66.30 ± 8.46	65.15 ± 7.02	70.63 ± 7.82	3.863	0.022
Disease duration, months	26.63 ± 23.64	28.33 ± 26.56	41.00 ± 39.30	1.603	0.208
Male sex, n(%)	12 (46.2)	15 (53.6)	15 (50.0)	0.297	0.862
Education, years	11.67 ± 2.71	9.85 ± 3.53	9.07 ± 4.39	4.033	0.028
MMSE score	28.04 ± 1.34	25.41 ± 2.56	20.77 ± 3.61	51.159	<0.001
MoCA score	24.96 ± 2.21	19.89 ± 2.86	14.80 ± 3.73	84.445	<0.001
ADL score	24.11 ± 9.46	27.22 ± 9.46	34.67 ± 12.99	7.432	0.001
HAMD score	8.96 ± 4.76	9.70 ± 5.25	10.40 ± 6.43	0.425	0.655
HAMA score	13.00 ± 4.94	13.44 ± 4.93	13.73 ± 6.28	0.089	0.915
Hoehn and Yahr stage	1.93 ± 0.73	2.15 ± 0.85	2.48 ± 0.85	5.128	0.038
UPDRS-III score	26.15 ± 12.62	30.78 ± 14.27	38.77 ± 12.95	6.937	0.002
BMI, kg/m^2^	24.63 ± 3.06	24.39 ± 4.01	23.69 ± 2.87	0.648	0.760

Values are mean ± standard deviation unless otherwise indicated.

### 3.2 Sleep architecture and nocturnal ECG parameters across cognitive impairment stages

Macro-sleep indicators, including total bed time, sleep efficiency, absolute sleep latency, percentage of slow-wave sleep, and baseline severity of sleep-disordered breathing (indexed by AHI), did not manifest distinct variances across the cohorts (all *p* > 0.05). However, fine-grained neurophysiological and ECG parameters varied significantly. Post-hoc analysis showed that the REM sleep latency was significantly shortened in both the PD-MCI (123.06 ± 74.48 min) and PDD (128.82 ± 117.32 min) arms relative to the PD-NC reference cohort (200.54 ± 90.26 min, *F* = 4.464, *p* = 0.006). Furthermore, the mean nocturnal heart rate during sleep was significantly accelerated in the PDD group (65.80 ± 10.80 bpm) compared to the PD-MCI (59.04 ± 6.63 bpm) and PD-NC (59.44 ± 5.54 bpm) cohorts (*F* = 6.365, *p* = 0.003). A parallel significant elevation was observed for the minimum nocturnal heart rate (*F* = 4.757, *p* = 0.008; Table 2).

**Table 2:** PSG parameters and nocturnal heart rate indices Across Cognitive Groups.

Variable	PD-NC (*n* = 27)	PD-MCI (*n* = 27)	PDD (*n* = 30)	*F*	*p*
Total bed time (min)	537.04 ± 68.73	550.59 ± 90.51	539.28 ± 103.05	0.275	0.835
Total sleep time (min)	360.63 ± 116.19	398.52 ± 102.66	359.95 ± 94.29	1.349	0.298
Sleep efficiency (%)	66.40 ± 15.66	71.80 ± 16.12	67.11 ± 14.06	1.021	0.369
Sleep latency (min)	27.87 ± 44.64	24.57 ± 21.72	21.05 ± 16.73	0.459	0.691
REM sleep latency (min)	200.54 ± 90.26	123.06 ± 74.48	128.82 ± 117.32	4.464	0.006
Stage I sleep duration (min)	53.93 ± 27.86	52.30 ± 30.22	71.45 ± 47.85	2.381	0.098
Stage I sleep (%)	16.39 ± 10.39	14.56 ± 9.81	21.17 ± 16.24	2.074	0.130
Stage II sleep duration (min)	209.31 ± 89.75	228.56 ± 77.75	190.07 ± 75.57	1.501	0.208
Stage II sleep (%)	56.23 ± 15.77	59.04 ± 12.13	53.35 ± 18.14	0.814	0.394
Slow-wave sleep duration (min)	51.04 ± 46.69	44.37 ± 41.52	48.07 ± 55.29	0.014	0.880
Slow-wave sleep (%)	14.58 ± 12.38	11.05 ± 10.74	12.45 ± 13.89	0.283	0.579
REM sleep duration (min)	48.98 ± 35.25	62.28 ± 30.68	48.35 ± 35.09	1.410	0.231
REM sleep (%)	12.79 ± 7.74	15.34 ± 5.88	13.02 ± 8.82	0.840	0.396
Apnea-hypopnea index (AHI)	4.98 ± 7.01	4.85 ± 6.39	7.38 ± 7.46	1.254	0.308
Hypopnea index	5.61 ± 4.38	7.53 ± 9.25	6.83 ± 6.90	0.620	0.608
Time with SpO₂ < 90% (%)	2.92 ± 2.92	1.92 ± 3.26	1.19 ± 2.38	0.858	0.408
Mean nocturnal heart rate (bpm)	59.44 ± 5.54	59.04 ± 6.63	65.80 ± 10.80	6.365	0.003
Maximum nocturnal heart rate (bpm)	91.85 ± 16.59	96.59 ± 16.84	97.90 ± 24.39	0.729	0.490
Minimum nocturnal heart rate (bpm)	44.15 ± 10.19	40.93 ± 12.85	50.77 ± 12.51	4.757	0.008
PLMS index	2.35 ± 1.45	2.43 ± 1.45	2.90 ± 1.81	1.098	0.372
Wake time after sleep onset (min)	142.52 ± 74.16	112.56 ± 91.33	143.35 ± 75.78	1.105	0.277

Values are mean ± standard deviation. PLMS, periodic limb movements during sleep.

### 3.3 Non-cognitive parameters correlated with cognitive deficit stages

Spearman correlation analysis showed that higher cognitive impairment stage was associated with higher age, Hoehn and Yahr stage, UPDRS-III score, ADL score, frequency of mixed apnea episodes during REM sleep, mean nocturnal heart rate, and minimum nocturnal heart rate. Higher education and longer REM sleep latency were inversely associated with cognitive impairment stage (Figure 1).

**Figure 1:**
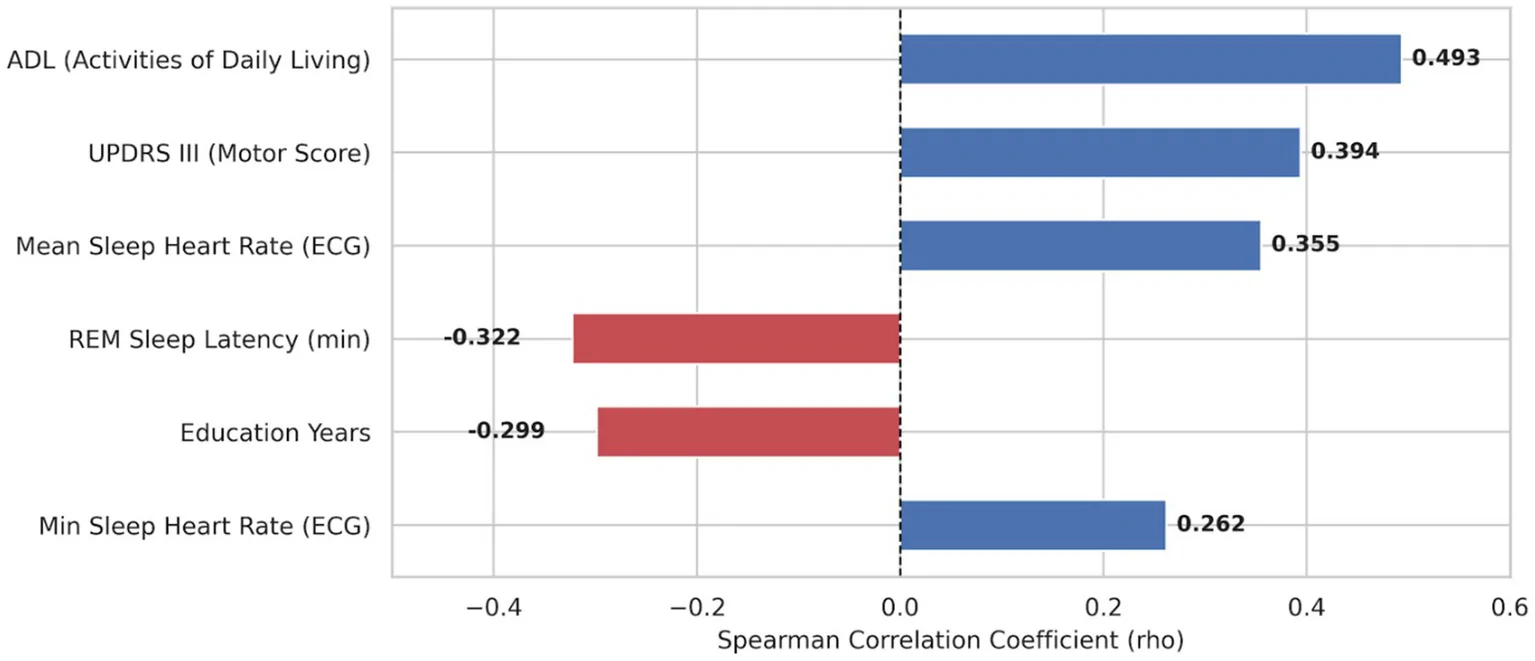
Clinical and PSG correlates of cognitive impairment severity category. Variables significantly associated with cognitive impairment severity in Spearman rank analyses (*p* < 0.05). ADL, activities of daily living; UPDRS-III, Unified Parkinson’s Disease Rating Scale Part III; ECG, electrocardiogram; REM, rapid eye movement.

### 3.4 Multivariable regression analysis of MoCA score

To evaluate independent associations with global cognition, a multivariable linear regression model was constructed using the continuous MoCA raw score as the dependent variable. The model was statistically significant (*R*^2^ = 0.438, adjusted *R*^2^ = 0.402, *F* = 12.14, *p* = 1.04 × 10^−^8). After adjusting for age, education, and motor severity, both nocturnal physiological variables remained independently associated with MoCA score: mean nocturnal heart rate was negatively associated with MoCA score (*B* = −0.158, *β* = −0.264, *t* = −3.018, *p* = 0.003), whereas REM sleep latency was positively associated with MoCA score (*B* = 0.010, *β* = 0.192, *t* = 2.224, *p* = 0.029). Collinearity diagnostics yielded VIF values of 1.029–1.261, indicating no important multicollinearity among included predictors (Table 3).

**Table 3:** Multivariable linear regression analysis with MoCA score as the dependent variable.

Variable	*B*	SE	*β*	*t*	*p*	95% CI	VIF
Constant	30.551	4.619	–	6.614	<0.001	21.355 to 39.747	–
Age	−0.090	0.059	−0.141	−1.519	0.133	−0.208 to 0.028	1.191
Education	0.549	0.125	0.400	4.403	<0.001	0.301 to 0.797	1.146
UPDRS-III score	−0.064	0.035	−0.175	−1.836	0.070	−0.133 to 0.005	1.261
REM sleep latency	0.010	0.004	0.192	2.224	0.029	0.001 to 0.018	1.029
Mean nocturnal heart rate	−0.158	0.052	−0.264	−3.018	0.003	−0.263 to −0.054	1.063

SE, standard error; CI, confidence interval; VIF, variance inflation factor.

The distinct linear dependency and patient cohort clustering for both key electrophysiological markers are graphically illustrated in Figure 2.

**Figure 2:**
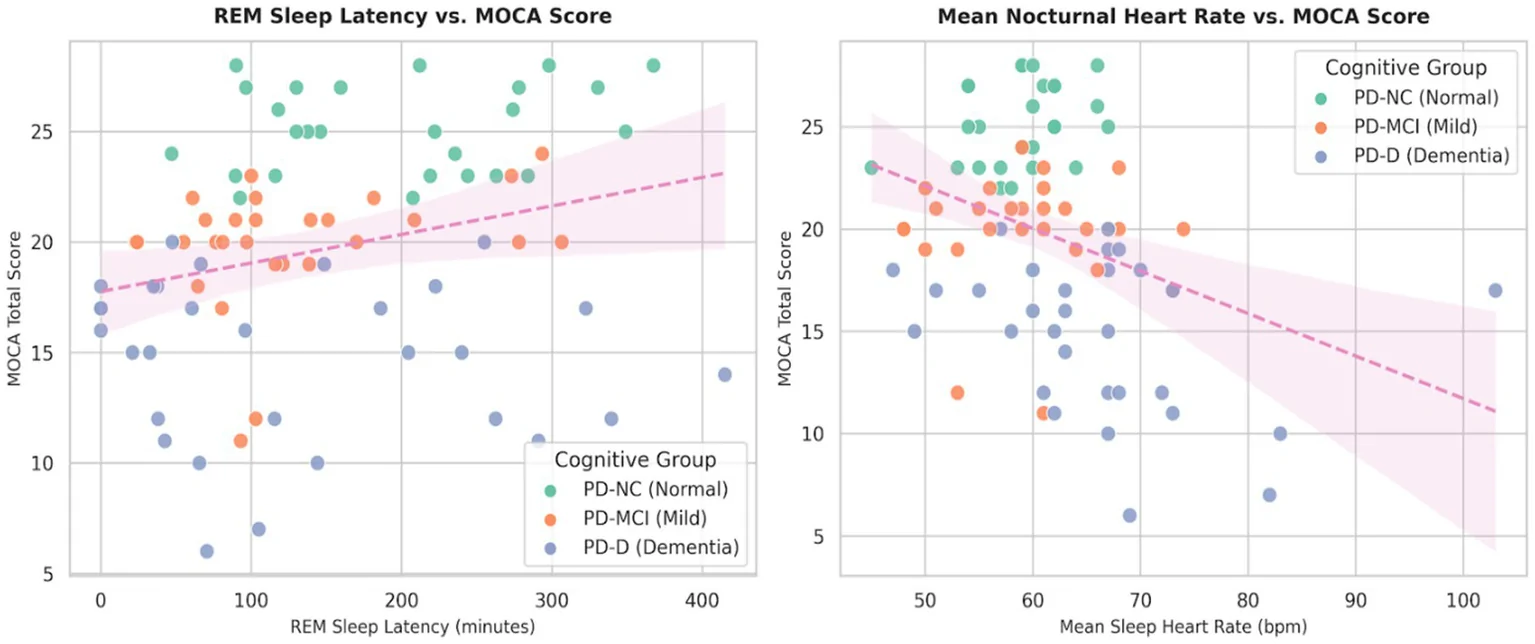
Scatter plots with linear regression lines illustrating the correlations between global cognitive function and core electrophysiological biomarkers. (Left) MoCA Score vs. REM Sleep Latency: longer REM latency was associated with higher MoCA scores across the cohort. (Right) MoCA Score vs. Mean Nocturnal Heart Rate: higher mean nocturnal heart rate was associated with lower MoCA scores. The plots illustrate unadjusted relationships and should not be interpreted as demonstrating causality. Dots represent the PD-NC, PD-MCI, and PDD groups; dashed lines indicate ordinary least-squares regression trends with 95% confidence intervals.

## 4 Discussion

This study evaluated associations between cognitive impairment in PD and specific overnight vPSG and heart-rate measures. In multivariable linear regression, higher mean nocturnal heart rate (*p* = 0.003) and shorter REM sleep latency (*p* = 0.029) remained associated with lower MoCA scores after adjustment for age, education, and UPDRS-III motor score. These observations support the potential value of nocturnal physiological measures as correlates of cognitive status. However, given the cross-sectional design and the absence of direct measures of autonomic balance or cholinergic function, the underlying mechanisms cannot be established by the present data.

### 4.1 Shortened REM sleep latency and possible cholinergic involvement

After adjustment for age, education, and motor severity, REM sleep latency remained positively associated with MoCA score (*B* = 0.010, *p* = 0.029). However, the group-level pattern was not consistent with a progressive linear change across cognitive stages. REM latency was shorter in both PD-MCI and PDD than in PD-NC, but was similar between the PD-MCI and PDD groups. The association observed in the full cohort may therefore reflect a difference between cognitively normal and cognitively impaired patients, rather than a gradual shortening of REM latency as cognitive impairment becomes more severe.

REM sleep is regulated by interacting brainstem circuits and neurotransmitter systems, including cholinergic pathways (10). Shortened REM sleep latency has previously been reported in patients with PD (20), and sleep-related electrophysiological abnormalities have been associated with subsequent cognitive decline (10). Although REM sleep behavior disorder is distinct from REM sleep latency, its association with cognitive dysfunction further supports the clinical relevance of REM-related disturbances in PD (21). These observations provide a possible biological context for the association identified in our study but do not establish a cholinergic mechanism. We did not directly assess cholinergic integrity using molecular imaging, neurochemical biomarkers, or neuropathological measures. Shortened REM latency should therefore not be regarded as a direct marker of cholinergic denervation on the basis of the present data. Whether cholinergic dysfunction contributes to this association requires confirmation in longitudinal studies incorporating direct measures of cholinergic function.

### 4.2 Elevated nocturnal heart rate and possible alterations in cardiac autonomic regulation

Mean nocturnal heart rate was negatively associated with MoCA score after multivariable adjustment (*β* = −0.264, *p* = 0.003). However, mean heart rate was similar in the PD-NC and PD-MCI groups and was substantially higher only in the PDD group. The association observed in the full cohort may therefore have been driven mainly by the PDD group rather than representing a uniform linear relationship across the cognitive spectrum.

Cardiac autonomic dysfunction is a recognized non-motor feature of PD (11), and previous studies using HRV have reported alterations in cardiac autonomic regulation in relation to PD and cognitive impairment (12, 22). Nevertheless, mean heart rate is a nonspecific measure. It cannot distinguish sympathetic from parasympathetic contributions and may be influenced by medications, respiratory events, cardiovascular disease, arousals, and sleep-stage distribution. The present result should therefore be interpreted as an association between a PSG-derived ECG measure and cognitive performance, rather than direct evidence of sympathetic predominance or sympathovagal imbalance.

### 4.3 Cognitive reserve and covariate influences

Consistent with existing literature, years of formal education emerged as a significant positive predictor in our regression model, in line with the cognitive reserve hypothesis (23). After multivariable adjustment, UPDRS-III motor score showed a trend toward association (*p* = 0.070), whereas age was not statistically significant (*p* = 0.133). These results indicate that the associations of REM latency and nocturnal heart rate with MoCA score were not explained by the covariates included in this model; they do not exclude residual confounding or establish PD-specific causal mechanisms.

Furthermore, while chronological age and disease duration were controlled for in our multivariable model—thereby capturing much of the variance associated with age at onset—the inherent biological distinctiveness between early-onset and late-onset PD warrants consideration. Late-onset PD is typically associated with faster progression of non-motor symptoms, including accelerated central cholinergic denervation and autonomic failure. Future studies stratified specifically by age at onset are encouraged to evaluate whether these nocturnal markers display differential sensitivity across clinical subphenotypes.

## 5 Limitations

Several limitations should be considered. First, the cross-sectional, single-center design and modest sample size limit the generalizability of the findings and preclude conclusions about causality or temporal relationships. Second, cognition was assessed using the global MoCA score under the MDS Level I framework. A comprehensive Level II neuropsychological battery would be needed to determine whether the observed associations relate to specific cognitive domains or to overall cognitive impairment. Third, patients were not stratified by age at PD onset, and possible differences between early and late onset PD could not be examined. Fourth, cardiac autonomic regulation and central cholinergic integrity were not directly assessed. The cardiac analysis was limited to whole-night mean, minimum, and maximum heart rates and did not include beat-to-beat HRV or sleep-stage-specific ECG measures. Similarly, REM sleep latency is not a direct measure of cholinergic function, and no cholinergic imaging or neurochemical biomarkers were available. Fifth, residual confounding by medication exposure cannot be excluded. Although patients taking sleep-modifying drugs were excluded, LEDD, individual dopaminergic regimens, medication timing relative to vPSG, and cardiovascular or autonomically active medications were not systematically recorded for all participants. Medication-adjusted and medication-stratified sensitivity analyses could therefore not be performed. Finally, the associations observed in the full cohort may have been influenced disproportionately by the PDD group, and the sample size limited further analyses of nonlinearity and sensitivity analyses restricted to patients without dementia. Larger multicenter longitudinal studies incorporating detailed medication data, domain-specific cognitive testing, beat-to-beat sleep-stage-specific HRV, and direct measures of cholinergic function are needed to confirm these findings.

## 6 Conclusion

In this cross-sectional cohort of 84 patients with PD, shorter REM sleep latency and higher mean nocturnal heart rate were independently associated with lower MoCA scores after adjustment for age, education, and motor severity. These measures may represent accessible nocturnal physiological correlates of cognitive status. Their mechanistic basis, longitudinal predictive value, and independence from medication and sleep-stage effects require confirmation using direct autonomic measures, stage-specific analyses, and prospective external validation.

## Data Availability

The raw data supporting the conclusions of this article will be made available by the authors, without undue reservation.
